# Radiomics-Based Prediction of Collateral Status from CT Angiography of Patients Following a Large Vessel Occlusion Stroke

**DOI:** 10.3390/diagnostics14050485

**Published:** 2024-02-23

**Authors:** Emily W. Avery, Anthony Abou-Karam, Sandra Abi-Fadel, Jonas Behland, Adrian Mak, Stefan P. Haider, Tal Zeevi, Pina C. Sanelli, Christopher G. Filippi, Ajay Malhotra, Charles C. Matouk, Guido J. Falcone, Nils Petersen, Lauren H. Sansing, Kevin N. Sheth, Seyedmehdi Payabvash

**Affiliations:** 1 Section of Neuroradiology, Department of Radiology and Biomedical Imaging, Yale School of Medicine, New Haven, CT 06520, USA; emilywavery@gmail.com (E.W.A.); adrian.mak@yale.edu (A.M.);; 2 CLAIM—Charité Lab for Artificial Intelligence in Medicine, Charité—Universitätsmedizin Berlin, 10117 Berlin, Germany; 3 Department of Otorhinolaryngology, University Hospital of Ludwig Maximilians Universität München, 81377 Munich, Germany; 4 Section of Neuroradiology, Department of Radiology, Donald and Barbara Zucker School of Medicine at Hofstra/Northwell Health, Manhasset, NY 11030, USA; 5 Section of Neuroradiology, Department of Radiology, Tufts School of Medicine, Boston, MA 02111, USA; 6 Division of Neurovascular Surgery, Department of Neurosurgery, Yale School of Medicine, New Haven, CT 06520, USA; 7 Division of Neurocritical Care and Emergency Neurology, Department of Neurology, Yale School of Medicine, New Haven, CT 06520, USA; 8 Division of Stroke and Vascular Neurology, Department of Neurology, Yale School of Medicine, New Haven, CT 06520, USA

**Keywords:** stroke, large vessel occlusion, radiomics, machine learning, collateral status

## Abstract

Background: A major driver of individual variation in long-term outcomes following a large vessel occlusion (LVO) stroke is the degree of collateral arterial circulation. We aimed to develop and evaluate machine-learning models that quantify LVO collateral status using admission computed tomography angiography (CTA) radiomics. Methods: We extracted 1116 radiomic features from the anterior circulation territories from admission CTAs of 600 patients experiencing an acute LVO stroke. We trained and validated multiple machine-learning models for the prediction of collateral status based on consensus from two neuroradiologists as ground truth. Models were first trained to predict (1) good vs. intermediate or poor, or (2) good vs. intermediate or poor collateral status. Then, model predictions were combined to determine a three-tier collateral score (good, intermediate, or poor). We used the receiver operating characteristics area under the curve (AUC) to evaluate prediction accuracy. Results: We included 499 patients in training and 101 in an independent test cohort. The best-performing models achieved an averaged cross-validation AUC of 0.80 ± 0.05 for poor vs. intermediate/good collateral and 0.69 ± 0.05 for good vs. intermediate/poor, and AUC = 0.77 (0.67–0.87) and AUC = 0.78 (0.70–0.90) in the independent test cohort, respectively. The collateral scores predicted by the radiomics model were correlated with (rho = 0.45, *p* = 0.002) and were independent predictors of 3-month clinical outcome (*p* = 0.018) in the independent test cohort. Conclusions: Automated tools for the assessment of collateral status from admission CTA—such as the radiomics models described here—can generate clinically relevant and reproducible collateral scores to facilitate a timely treatment triage in patients experiencing an acute LVO stroke.

## 1. Introduction

There is considerable variability in clinical outcomes and the extent of ischemic damage among patients experiencing a large vessel occlusion (LVO) stroke after an endovascular thrombectomy (EVT) [1]. One significant contributor to this variation is differing degrees of collateral arterial circulation beyond the site of occlusion, wherein patients with better collateral flow suffer less neurological damage and have better outcomes [2]. Indeed, patients with good collateral flow have demonstrated higher rates of recanalization after EVT or intravenous thrombolysis, lower final infarct volumes, a slower rate of infarct progression, and better functional outcomes than patients with poor collateral flow [3,4,5].

The recognition that collateral status affects LVO stroke outcomes after EVT is reflected by current guidelines and proposed prognostic scales [6]. Recent trials have recognized the added value of collateral assessment in early LVO ischemic changes, noting, critically, that poor collaterals indicate a need for rapid recanalization [7]. To this end, the effect of collateral status on the relative benefit of extended-time-window EVT (6-to-24 h after onset) remains an area of active research [8]. While a quantification of collateral status is required for its use as a universal prognostic biomarker, commonly used scales vary in both number of categories and type of assessment, and collateral status grading has yet to be standardized [9,10]. Collateral status scoring is also not routinely required in radiologists’ reports and is not an inherent part of the ‘code stroke’ CT angiography (CTA) workflow. As timely, accurate decision making in the acute stroke setting is imperative, a quick, objective, and automated assessment tool would be an ideal way to counter inconsistencies in collateral scoring and assist in time-sensitive patient triage.

In the present study, our overarching goal was to assist LVO stroke treatment decision making and expedite risk stratification in these patients upon admission based on collateral status. To this end, we hypothesized that the radiomic features of admission CTA scans could provide an objective measure of collateral arterial flow in patients experiencing an acute LVO stroke. Radiomics-based models, in which several hundred quantitative features derived from medical images are fed to machine learning algorithms for the prediction of a variable of interest, have already proven useful in predicting clinical variables that can assist with prognostication in acute LVO stroke cases [11,12,13,14]. We thus aimed to use radiomics methods to create an automated assessment platform for collateral flow. From a database of 600 acute LVO stroke patients, we extracted radiomic features from the anterior circulation territories of admission CTAs, and then, using multiple combinations of feature selection and machine learning classifiers, we trained, optimized, and validated models to quantify collateral arterial flow. The present work describes a methodology for the development and testing of these models, analyzes their performance in an independent test cohort, and evaluates the clinical relevance of their predictions as compared to collateral scoring determined by neuroradiologists.

## 2. Methods

### 2.1. Data Acquisition

From the Yale New Haven Hospital stroke center registry, 600 consecutive subjects who experienced an LVO stroke were identified between 1 January 2020–31 October 2020. Included patients met the following criterial: (1) all patients suffered an anterior circulation LVO stroke (ICA, M1, or M2 occlusion); (2) all patient had an admission CTA scan with slice thickness ≤1 mm; (3) all patients were sent for EVT intervention; and (4) the functional outcome for all patients was recorded at discharge and at 3-month follow-up (or the closest follow-up interval) using the modified Rankin Scale (mRS) functional outcome assessment. EVT reperfusion success was quantified by the treating neuro-interventionalist according to the modified Thrombolysis in Cerebral Infarction (mTICI) scale. Patients were excluded for the following reasons: (a) the patient had a simultaneous posterior circulation LVO; (b) the patient’s admission CTA was of poor quality because of motion, metal artifact, or scanner-based artifacts; or (c) the patient was missing admission or follow-up clinical information. We obtained approval from our institution’s institutional review board (IRB) for our study. Our IRB approval included a waiver of informed consent from study participants due to the retrospective nature of our data collection. Patient management and procedures at our hospital were followed according to the institutional and national guidelines at the time of patient admission and were not influenced by research protocols.

### 2.2. Collateral Status

For assessment of collateral status, we used the 3-point Miteff scoring system given its easy-to-use 3-level scoring [15], and proven reliability for predicting outcomes in thrombolyzed stroke patients compared to other scoring systems [16]. In this scoring system, a grade of 3 is assigned if vessels are reconstituted distal to the occlusion, a grade of 2 is assigned if vessels can be seen at the Sylvian fissure, or a grade of 1 is assigned when the contrast opacification is seen only in the distal superficial branches [15]. For each of the included subjects, collateral status scores were independently quantified by two neuroradiologists (S.P. and A.K.) as poor, intermediate, or good [15]. Both neuroradiologists were board certified, each with at least 7 years of experience in reviewing brain CTAs, and were blinded to each patient’s clinical and other imaging data. In subjects with disagreement, consensus scores were determined conjointly. We calculated interrater correlation using Cohen’s kappa and compared collateral status correlations with patient outcomes (mRS at 3 months) using Fisher’s r-to-z transformation. The two-rater consensus score was used as the measure of ground truth for collateral status modeling and analyses.

### 2.3. Image Pre-Processing and Feature Extraction

The radiomics features of middle cerebral artery (MCA) territories were extracted from native CTAs as previously described [17]. Image pre-processing was conducted as follows: isotropic 1-mm spacing of voxels spacing was achieved through image resampling to ensure rotational invariance of texture features. Given that intravenous bolus timing may differ during CTA scan acquisition, all images were normalized, and voxels were only included if they were between a 1-to-500 Hounsfield unit (HU) range. We extracted a total of 1116 “texture-matrix” and “first-order” radiomics features from the CTA MCA territories after high- and low-pass filters were implemented in each spatial direction (“coif-1” transformation of wavelets and “edge-enhancement” Laplacian of Gaussian (LoG) filter using sigma values of 6, 4, and 2 mm), using a customized Pyradiomics pipeline [18]. Radiomic features included first-order features (e.g., kurtosis, mean, variance), gray level co-occurrence texture features (e.g., contrast), gray level size zone texture features (e.g., gray level variance), gray level run length matrix features (e.g., run entropy), neighboring gray tone difference matrix features (e.g., coarseness), gray level dependence matrix features (e.g., gray level variance). Detailed descriptions of radiomic features are provided in Supplemental Table S1, and a complete list of the first-order and texture features used in this study is described in van Griethuysen et al., 2017 [19].

### 2.4. Training and Optimization of Models

Separate datasets were allocated for training/cross-validation and independent testing, with patient dataset assignment made at random. Separate models were trained for binary prediction of either (1) poor vs. intermediate or good collateral status, or (2) good vs. poor or intermediate collateral status. Following the methodological framework first described in Haider et al. [20], we applied 50-to-200 rounds of 5-fold cross-validation to optimize the hyperparameters for each machine-learning algorithm using Bayesian Optimization [21]. After optimization, 20 rounds of 5-fold cross-validation were performed using the optimized hyperparameters and the receiver operating characteristics (ROC) area under the curve (AUC) subsequently determined in validation folds for each combination of 6 feature selection methods and 6 machine learning classifiers (36 pairs). The average AUC across 100 validation folds was calculated and used to determine the optimal performing model (pair of feature selection method and machine-learning classifier). The 6 feature selection methods and 6 machine learning classifiers are described below. The machine learning classifiers’ hyperparameters and their range are specified in Supplemental Table S2. All 36 possible combinations of the six feature selection methods (A) and six machine learning classifiers (B) were used to create candidate models for prediction of collateral status. Detailed descriptions of each feature selection method and machine learning classifier are noted in previous work [20].

#### 2.4.1. Feature Selection Methods

Hierarchical clustering (HClust): In this feature selection method, we first computed an Euclidean feature distance matrix of all radiomic features using the “stats” package in R (version 3.6.0) [22]. This was followed by Ward clustering [23], and cutting the resultant dendrogram until 20 clusters remained, allowing for extraction of “meta-features” by averaging the features of the remaining clusters as 20 meta features in analysis.

Minimum redundancy maximum relevance filter (MRMR): Using the R “mRMRe” package (version 2.0.9) [24], we perform traditional MRMR feature selection to select the n most predictive features, as n was tuned during Bayesian optimization as a hyperparameter.

No feature selection (noFS): No feature selection was performed for this method. The classifiers were fitted on the entire feature set.

Principal component analysis (PCA): Using the “prcomp” function of the R “stats” package (version 3.6.0) [22], we adapted PCA for feature selection using the schemata proposed by Song et al. [25], wherein 30 eigenvectors were selected and ranked. The n features contributing the most to the feature extraction result were used for classifier fitting.

Pearson correlation-based redundancy reduction with mutual information maximization filter (pMIM): First, we computed Pearson’s correlation coefficient (r) for all radiomic feature pairs using the “cor” function of the R “stats” package (version 3.6.0) [22], and excluded feature pairs with an absolute r value > 0.9 to reduced multicollinearity (R “caret” package “findCorrelation” function) [26]. We then applied a mutual information maximization filter to non-redundant features using “MIM” function of “praznik” package (version 6.0.0) R [27].

RIDGE regularized logistic regression for feature selection (RIDGE): We used the R “glmnet” package (version 2.0-18) [28] “cv.glmnet” function to fit a ridge regularized logistic regression model. The lambda parameter was determined using the cv.glmnet function’s internal 10-fold cross-validation. Each feature’s regression coefficient was derived from the fitted “glmnet” at a lambda value that maximized the mean cross-validated AUC. Then, the n highest-ranked features based on absolute regression coefficient were selected.

#### 2.4.2. Machine Learning Classifiers

Elastic net regularized logistic regression (ElNet): We used the “cv.glmnet” function of the R “glmnet” package (version 2.0-18) [28]. The lambda parameter was determined using the internal 10-fold cross-validation mode of the “cv.glmnet” function similar to the Ridge model. The Elastic Net regression provides a hybrid approach that blends both penalizations of the L2 and L1 regularization of lasso (alpha = 1) and Ridge (alpha = 0) methods. We finetuned the alpha hyperparameter during Bayesian optimization process.

Naïve Bayes classifier (NBayes): For this machine learning classifier, we used the “naive_bayes” function of the R “naivebayes” package (version 0.9.6) [29] to create the models. We did not use Laplace smoothing or kernel.

Random forest classifier (RF): We used the R “randomForest” package (version 4.6-14) [30], and configured the model to grow 1000 trees and perform sampling of cases with replacement. The “mtry” parameter (the number of features randomly sampled at each split) and the “maxnodes” parameter (the maximum number of terminal nodes in a tree) were tuned in Bayesian optimization. All other function parameters were kept at their default values.

Support vector machine classifier, (SVM_sig) and (SVM_rad): We used the R “e1071” (version 1.7-2) package [31] to implement SVM with “radial” and “sigmoid” kernels. Sigmoid and radial kernels are among the most widely used SVM kernels. The sigmoid kernel is most commonly used as a proxy for neural networks. The radial kernel is a general-purpose kernel that is appropriate for use when there is no prior knowledge about the data [32]. In SVM with radial kernel (SVM_rad), the “gamma” and “cost” parameters were optimized. In SVM with sigmoid kernel (SVM_sig), the “gamma”, “coef0”, and “cost” parameters were optimized. Class weights were specified to be inversely proportional to the class distribution in the training data, and all other parameters were kept at default values.

Extreme gradient boosting classifier (XGB): For this machine learning classifier, we implemented extreme gradient boosting utilizing the R “xgboost” package [33,34] in tree-booster mode (the “gbtree” option). We tuned “eta”, “gamma”, “max_depth”, “subsample”, “lambda”, “min_child_weight”, and “colsample_bytree” with 300 iterations of boosting. The remaining parameters were kept at their default values. 

### 2.5. Final Model Training and Validation

For independent validation, we identified the candidate model (feature selection method and machine learning algorithm pair) with the highest averaged cross-validation AUC. Then, we trained this model on the complete training/cross-validation dataset applying optimized machine learning hyperparameters. This final model was then applied to the independent test cohort to predict collateral status. The independent test cohort was completely isolated from the training/cross-validation process. We used DeLong’s test to evaluate paired AUCs and to calculate the *p*-value and 95% confidence interval (CI) for each AUC using the R pROC package [35,36]. We also used the multi-class ROC analysis from the pROC package, to compare the accuracy of three-tier collateral status prediction versus consensus scores in independent test cohort. The multiclass AUC is the mean of separate AUCs and cannot be plotted. Similarly, confidence intervals, standard deviation, smoothing and comparison tests are not applicable to this analysis [37]. 

### 2.6. Statistical Methods

For univariate comparison between the training/cross-validation and independent test groups, we used the Student’s *t*-test for continuous variables, the Mann–Whitney rank test for ordinal variables, and the Fisher exact test for categorical variables. We used Spearman rank correlation to determine the relationship between collateral status ratings and predicted collateral scores with patient 3-month mRS outcome. We also performed multivariate ordinal logistic regression to determine whether collateral scores were independent predictors of outcome—adjusting for age, sex, admission NIHSS, and post-EVT reperfusion mTICI scores. The threshold for statistical significance was a *p* value < 0.05.

## 3. Results

### 3.1. Patients Characteristics

A total of 600 patients were included in our analysis. The demographic characteristics of the training/cross-validation (*n* = 499) and independent test cohorts (*n* = 101) are detailed in Table 1. Between these two groups, there was no significant difference in average age, sex, admission NIHSS, onset-to-imaging time, or functional outcome at 3 months (Table 2). The consensus collateral scores were good, intermediate, and poor in 212 (42%), 174 (35%), and 113 (23%) patients in the training cohort, and 37 (37%), 35 (35%), and 29 (29%) patients in the test cohort, respectively (score distribution difference not significant, *p* = 0.17).

### 3.2. Comparison of Different Model Combinations for Predicting Collateral Status

**Figure 1:**
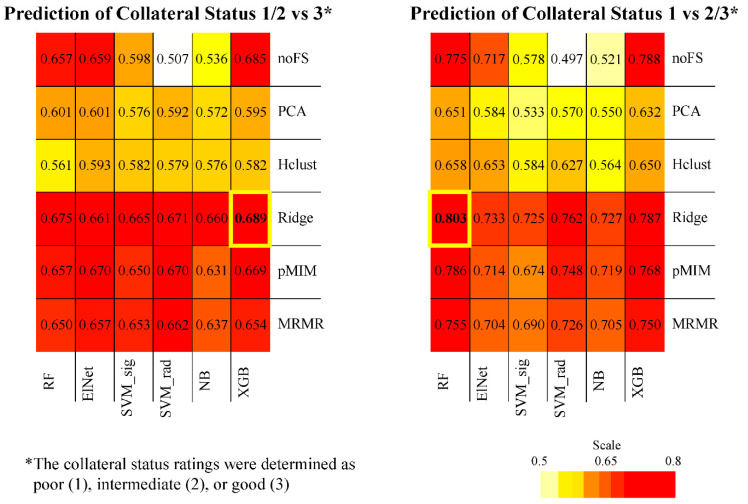
Heatmap summary of cross-validation performance for all candidate models. The feature selection/machine-learning combinations with the highest averaged area under the curve (AUC) across validation folds (from 20 repeats × 5-fold cross-validation) are highlighted with bold yellow cell border lines.

A heatmap summary of the performance of all collateral status candidate models in cross-validation is provided in Figure 1. The highest averaged AUC was 0.69 ± 0.05 for good vs. poor or intermediate collateral status prediction by combination of Ridge feature selection and random forest machine learning, and was 0.80 ± 0.05 for poor vs. intermediate or good collateral status prediction by combination of Ridge feature selection and XG-boost machine learning.

### 3.3. Independent Testing

**Figure 2:**
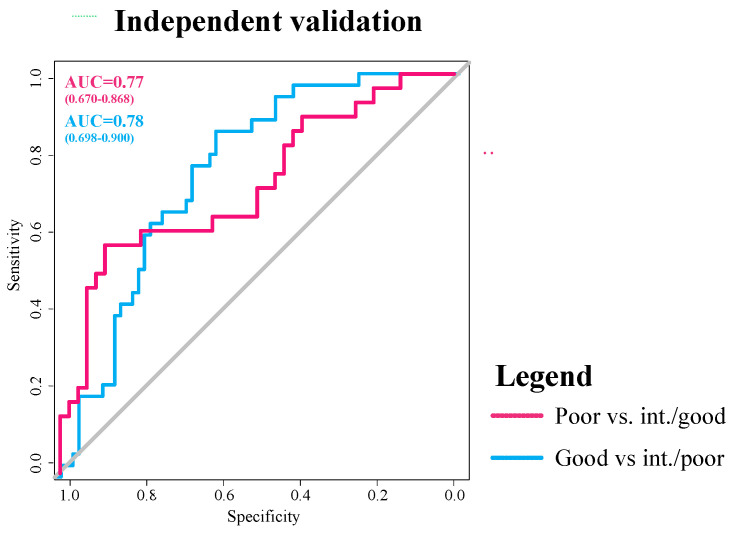
Receiver operating characteristics (ROC) area under the curve (AUC) analysis for collateral status prediction in the independent test cohort using Ridge feature selection and XG-boost classifier. ROC curves for predicting poor vs. intermediate or good flow are depicted in dotted line, and for predicting good vs. poor or intermediate flow are depicted in dashed line.

In the independent testing cohort, the Ridge and XG-boost combination achieved an AUC of 0.77 (0.67–0.87) for the prediction of poor vs. intermediate or good flow and an AUC of 0.78 (0.70–0.90) for good vs. poor or intermediate flow (Figure 2). Of note, the Ridge and random forest combination model had an AUC of 0.68 (0.57–0.79) for good vs. poor or intermediate flow, which was lower than the runner-up combination of Ridge and XG-boost in the cross-validation platform (Figure 1). On the independent test set, we also evaluated the accuracy of the three-tiered model predictions versus consensus scores (poor vs. intermediate vs. good) using multi-class ROC analysis, with a resulting multi-class AUC of 0.635 (notable, no 95% CI or *p* value is appropriate for this analysis).

### 3.4. Concordance Analysis

**Figure 3:**
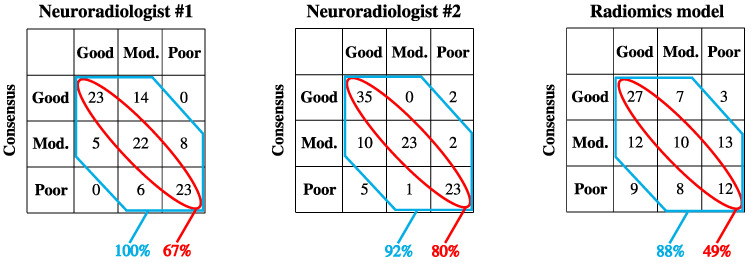
Concordance of collateral status scores by neuroradiologists and radiomics model predictions in independent test cohort (*n* = 101). Consensus scores were based on neuroradiologists #1 and #2’s ratings and consensus read on subjects with discrepancy. Ratings by neuroradiologist #1 were in agreement with the consensus score in 67% of cases, and were within one degree of the consensus score in 100% of cases. Ratings by neuroradiologist #2 were in agreement with the consensus score in 80% of cases, and were within one degree of the consensus score in 92% of cases. Ratings by the radiomics model were in agreement with the consensus score in 49% of cases, and were within one degree of the consensus score in 88% of cases.

By combining the predictions made by Ridge and XG-boost models in the independent test cohort, a single prediction of poor, intermediate, or good collateral flow was deduced. The concordance of radiomics model predictions with the consensus scores from both neuroradiologists are shown in Figure 3. The radiomics models predicted collateral status in agreement with consensus scores 49% of the time, with a Cohen’s kappa of 0.22 indicating fair concordance with the set of consensus scores [38]. By comparison, the neuroradiologists had an interrater concordance of kappa = 0.38, also indicating fair concordance.

### 3.5. Relationship of Collateral Score with Clinical Outcomes

In the independent test cohort, there was a significant correlation of 3-month outcome (0-to-6 mRS score) with consensus collateral scores (rho = 0.31, *p* = 0.002), collateral status as per neuroradiologist #1 (rho = 0.35, *p* < 0.001), collateral status as per neuroradiologist #2 (rho = 0.28, *p* = 0.005), and collateral status predicted by the radiomics model (rho = 0.45, *p* < 0.001). In the multivariate ordinal logistic regression, the collateral scores determined by neuroradiologist #1 (odds ratio = 1.64 (1.12–2.40), *p* = 0.012), and those predicted by the radiomics model (odds ratio = 2.38 (1.16–4.86), *p* = 0.018) were independently associated with 3-month mRS after adjusting for age, sex, admission NIHSS, and post-EVT reperfusion mTICI (Table 1).

In separate multivariate ordinal logistic regressions within independent test cohort (*n* = 101), the collateral status scores determined by neuroradiologist #1 and from radiomics model prediction were independent predictors of modified Rankin scale (mRS) at 3 months after adjustment for age, sex, NIHSS on admission, and post-thrombectomy reperfusion indicated by modified Thrombolysis in Cerebral Infarction score, mTICI.

## 4. Discussion

Using radiomic features extracted from the admission CTAs of acute LVO stroke patients, we devised, optimized, and validated machine learning classifiers to predict collateral status. In the independent test cohort, the collateral scores predicted by the radiomics model had significant correlations with clinical outcome and were independent predictors of outcome in multivariate regression. Our findings highlight the feasibility and clinical reliability of automated image analysis tools for the assessment of collateral status. Specifically, for subjective ratings such as collateral status, which lack objective gold standards, automated image analysis tools with reproducible and clinically relevant results can facilitate timely prognostication and treatment triage in the acute stroke setting.

One of the main challenges in training machine learning models for the prediction of collateral status is the subjective nature of the scoring system. In other words, the ground truth used for training and testing of the models is prone to interrater variability. Even after a one-hour dedicated training session with collateral score examples, the interclass correlation coefficient between 29 radiologists and radiology residents reached 0.75, using the Tan scale for collateral status [39,40]. We utilized the Miteff scale, as it has been a reliable scoring system for predicting both favorable and unfavorable outcome, rather than unfavorable outcome alone [16]. Nevertheless, the significant association of radiomics-generated collateral scores with 3-month mRS outcome in the independent test cohort (Table 1) is strong evidence for the clinical relevance of model prediction despite lower concordance with consensus scores compared to neuroradiologists (Figure 3).

A few groups have also developed automated tools for the assessment of collateral status. In 2023, Kuang and colleagues utilized a convolutional neural network (CNN) to predict collateral status in 154 patients experiencing an acute ischemic stroke using a three-tier scale for assessment of collateral status on single-phase CT and Maximum Intensity Projection (MIP) images for training and cross-validation. Binary prediction (good vs. poor collateral flow) AUCs for their cross validation models ranged from 0.71–0.79, comparable to our cross validation performance of 0.70–0.80 [41]. However, their study lacked an independent test cohort, and the best performing model in five-fold cross-validation achieved a 69% concordance with ground truth consensus scores from two neuroradiologists (scheme similar to Figure 3, wherein our model had a 49% concordance in a separate independent test dataset) [41]. In 2022, Wolff et al. [39] also utilized a CNN for collateral status scoring based on the four-tiered Tan scale [40] (absent collateral supply, >0% and ≤50% collateral supply, >50% and <100%, and 100%) using patients from the MR CLEAN registry [42]. However, concordance with radiologist-based consensus scores was reduced to a two-tier framework (good versus poor) in a random subset of patients, wherein it was found to be 59%. In addition, radiologist ratings that differed by more than one degree from the consensus were rescored by the first and senior authors, which might have improved interrater agreement [39]. 

Commercial software, such as e-CTA by Brainomix, has also been assessed for its potential to improve interrater concordance of quantitative collateral status scoring. In 2023, Jabal et al. [43] reported an improvement from 59% to 68% in interrater concordance when 12 raters (junior neuroradiologists, senior neuroradiologists, and neurologists) utilized e-CTA to assist in their ratings (four-tiered Tan scale) in cohort of 56 patients. However, the performance of the e-CTA tool itself and its concordance with the three senior raters who determined the consensus scores as well as the interrater concordance among the three senior raters were not reported [43]. In addition, there was no reported information regarding potential correlation between e-CTA collateral status and clinical outcome. The RAPID software also offers the hypoperfusion index ratio (HIR) to assess collateral status on multiphase CTA. However, recent comparisons of RAPID HIR with neuroradiologist collateral status ratings have been limited to a two-tier good vs. poor framework [44].

Compared to prior reports, the main strengths of our study are model validation in an independent test cohort; the use of three-tier collateral classification; the establishment of clinical correlation and relevance of model-predicted collateral scores; and the transparent depiction of concordance between the model and neuroradiologists with consensus collateral scores which were used as the ground truth. Automated tools for predicting collateral status allow for a more personalized approach to stroke management, considering the specific needs and potential responses of each patient. More consistent automated assessments of collateral status compared to visual evaluations can reduce inter-institutional variabilities in treatment decisions and research collaboration. Finally, automated assessment of the collateral status can help prioritize patient treatment based on those who have a more urgent need for reperfusion therapies.

The present work lays a foundation for the future implementation of fully automated assessment tools for LVO stroke patients. Such tools can ideally be implemented into stroke patient workflow such that the patient’s admission CTA scan is automatically segmented, radiomics features are automatically extracted, and these features are automatically analyzed as in the present work to derive a predicted quantitative measure of collateral flow. This requires no manual segmentation or analysis on behalf of the physician. In the ideal real-world setting, the radiologist, neurologist, and other consulting physicians can see and consider the predicted collateral status variables as they make time-sensitive treatment decisions. This streamlined, automated platform would prove to be especially valuable in tele-stroke settings, and in rural or underserved community hospitals. 

An important limitation of our study is the absence of LVO patients without thrombectomy treatment. Since collateral status has predominantly been addressed in the context of treatment guidance in thrombectomy candidates, we limited our study cohort to patients with LVO who underwent thrombectomy. This may have affected the proportions of good, intermediate, and poor collateral statuses in our study cohorts compared to all LVO stroke patients regardless of treatment status. The generalizability of our models to other institutions and patient populations should also be assessed in future work. Inconsistency in contrast administration and acquisition protocols across different centers is a crucial factor hampering reproducibility of collateral status assessment across different imaging studies. All patients in our study cohort had single-phase early arterial CTA acquisition; however, multiphase or late-phase acquisitions can inherently affect collateral status evaluation. Also, many other machine learning models such as Bayesian Network Classifier, AdaBoost Classifier, and Neural Network Classifiers were not tested in our analyses. Another limitation of our dataset is the fact that there were proportionally more M1 occlusions in the training cohort and proportionally more M2 occlusions in the testing cohort. In general, collateral flow becomes negligible and clinically irrelevant at the distal endpoints of an arterial tree [45]. However, precise differences in M1 vs. M2 collateral flow have not been extensively described, and do not likely make a clinically relevant difference in our study. There was also a proportionally higher rate of successful reperfusion in the testing cohort. As recent work has suggested that clinical outcome predicted by collateral flow is most reliable when EVT is technically successful [46], our analysis of the relationship between predicted collateral flow and functional clinical outcome (Table 1) is appropriately conducted within the testing cohort. In addition to the future study directions noted above, further work may also aim to improve the model’s performance by incorporating clinical data. Clinical variables such as smoking history [47], history of prior transient ischemic attack [48], and prior statin use [49], for example, have been associated with better collateral flow and may improve model performance. Patient age has conversely been associated with decreased collateral flow [50], though other clinical risk factors for stroke in very old age (>85) are thought to be unique [51] and future studies on this subset of patients alone is warranted. Overall, as clinical data are not always readily available in the time-sensitive period of ‘code stroke’ decision making, the fact that models based on imaging data alone demonstrate good performance remains important for real-life clinical scenarios.

## 5. Conclusions

In summary, our work illustrates that radiomics-based tools can be feasibly employed for automatically and objectively quantifying clinically relevant collateral statuses from admission CTA scans of patients experiencing an acute LVO stroke. In the test cohort, the scores predicted by our radiomics model were independent predictors of 3-month outcomes. As collateral status becomes an increasingly important clinical variable for prognostication and treatment decision-making in acute LVO stroke cases, automated models such as ours are particularly helpful given the lack of standardized collateral status scoring. To improve patient selection and, ultimately, patient outcomes, these tools may play a transformative role in facilitating time-sensitive and objective patient evaluation.

## Figures and Tables

**Table 1:** Evaluation of collateral scores as independent predictor of 3-month outcomes in multivariate model (test cohort, *n* = 101).

Collateral Status Score	Odds Ratio (95% Confidence Interval)	*p* Value
Neuroradiologist #1	1.64 (1.12–2.40)	0.012
Neuroradiologist #2	1.14 (0.75–1.72)	0.532
Consensus scores	1.61 (0.99–2.61)	0.054
Radiomics model prediction	2.38 (1.16–4.86)	0.018

**Table 2:** Training and testing cohort demographic characteristics.

	Training (*n* = 499)	Test (*n* = 101)	*p* Value
Patients’ age at admission (years)	70.4 ± 15.4	69.2 ± 14.0	0.471
Basline NIH Stroke Scale	15 (10–19)	13 (7–19.25)	0.126
Time gap from onset to angiography (hours)	7.2 ± 5.2	6.8 ± 4.8	0.501
Time gap from onset to CTA (hours)	5.3 ± 5.4	5.6 ± 5.3	0.603
Female sex	230 (46%)	48 (48%)	0.653
Side of occlusion side (right)	243 (48%)	41 (41%)	0.518
Internal Carotid Artery occlusion	120 (24%)	19 (19%)	0.254
Middle Cerebral Artery M1/M2 occlusion	379 (76%)	82 (81%)	0.254
modified Rankin Scale (mRS) score at 3-month	4 (2–6)	3 (1–6)	0.063
Favorable outcome at 3 months (mRS ≤ 2 at 3-month)	376 (75%)	68 (68%)	0.140

## Data Availability

All data are available from the corresponding author upon reasonable request.

## Supplementary Materials

The following supporting information can be downloaded at: https://www.mdpi.com/article/10.3390/diagnostics14050485/s1, Table S1: List of first-order and texture-based features; Table S2: Machine-learning classifiers’ hyperparameters and ranges.
