# Supplementary material for: Radiomics-Based Prediction of Collateral Status from CT Angiography of Patients Following a Large Vessel Occlusion Stroke
Source: Diagnostics (Basel). 2024 Feb 23;14(5):485. doi: 10.3390/diagnostics14050485 (PMC10930945; doi:10.3390/diagnostics14050485)
Supplement: Supplementary file 1 [file diagnostics-14-00485-s001.zip › diagnostics-2843178-supplementary.pdf]

Supplementary Table 1. List of first-order and texture-based features

**First-order Features**	
1	10th percentile
2	90th percentile
3	Energy
4	Entropy
5	Interquartile Range
6	Kurtosis
7	Maximum
8	Mean
9	Mean Absolute Deviation
10	Median
11	Minimum
12	Range
13	Robust Mean Absolute Deviation
14	Root Mean Squared
15	Skewness
16	Total Energy
17	Uniformity
18	Variance
**Texture - Gray Level Cooccurrence Matrix Features**	
1	Autocorrelation
2	Cluster Prominence
3	Cluster Shade
4	Cluster Tendency
5	Contrast
6	Correlation
7	Difference Average
8	Difference Entropy
9	Difference Variance
10	Informational Measure of Correlation 1
11	Informational Measure of Correlation 2
12	Inverse Difference
13	Inverse Difference Moment
14	Inverse Difference Moment Normalized
15	Inverse Difference Normalized
16	Inverse Variance
17	Joint Average
18	Joint Energy
19	Joint Entropy
20	Maximal Correlation Coefficient

21	Maximum Probability
22	Sum Average
23	Sum Entropy
24	Sum of Squares
**Texture - Gray Level Size Zone Matrix Features**	
1	Gray Level Non-Uniformity
2	Gray Level Non-Uniformity Normalized
3	Gray Level Variance
4	High Gray Level Zone Emphasis
5	Large Area Emphasis
6	Large Area High Gray Level Emphasis
7	Large Area Low Gray Level Emphasis
8	Low Gray Level Zone Emphasis
9	Size Zone Non-Uniformity
10	Size Zone Non-Uniformity Normalized
11	Small Area Emphasis
12	Small Area High Gray Level Emphasis
13	Small Area Low Gray Level Emphasis
14	Zone Entropy
15	Zone Percentage
16	Zone Variance
**Texture - Gray Level Run Length Matrix Features**	
1	Gray Level Non-Uniformity
2	Gray Level Non-Uniformity Normalized
3	Gray Level Variance
4	High Gray Level Run Emphasis
5	Long Run Emphasis
6	Long Run High Gray Level Emphasis
7	Long Run Low Gray Level Emphasis
8	Low Gray Level Run Emphasis
9	Run Entropy
10	Run Length Non-Uniformity
11	Run Length Non-Uniformity Normalized
12	Run Percentage
13	Run Variance
14	Short Run Emphasis
15	Short Run High Gray Level Emphasis
16	Short Run Low Gray Level Emphasis
**Texture - Neighboring Gray Tone Difference Matrix Features**	

1	Busyness
2	Coarseness
3	Complexity
4	Contrast
5	Strength
**Texture - Gray Level Dependence Matrix Features**	
1	Dependence Entropy
2	Dependence Non-Uniformity
3	Dependence Non-Uniformity Normalized
4	Dependence Variance
5	Gray Level Non-Uniformity
6	Gray Level Variance
7	High Gray Level Emphasis
8	Large Dependence Emphasis
9	Large Dependence High Gray Level Emphasis
10	Large Dependence Low Gray Level Emphasis
11	Low Gray Level Emphasis
12	Small Dependence Emphasis
13	Small Dependence High Gray Level Emphasis
14	Small Dependence Low Gray Level Emphasis

A complete list of the first-order and texture features used in this study is described in van Griethuysen et al., 2017[5], and exact feature definitions are described in Pyradiomics documentation[6].

Supplementary Table 2. Machine-learning classifiers' hyperparameters and ranges

ML classifier	n Rounds [a]	Hyperparameter [b]	Lower bound	Upper bound
ElNet[7]	100	n features [c]	2	30
		alpha	0	1
NBayes[8]	50	n features [c]	2	30
RF[9]	150	n features [c]	2	30
		mtry	2	40
		maxnodes	2	32768 [d]
SVM_rad[10]	150	n features [c]	2	30
		gamma	0	0.5
		cost	0.1	10
SVM_sig[10]	200	n features [c]	2	30
		gamma	0	0.5
		coef0	0	1
		cost	0.1	10
XGB[11, 12]	200	n features [c]	2	30
		eta	0	1
		gamma	0	10
		max_depth	3	15
		min_child_weight	0	20
		subsample	0.4	1
		colsample_bytree	0.4	1
		lambda	0.5	1

For each hyperparameter of each machine-learning classifier used in functional outcome prediction models, the number of optimization rounds performed, upper bounds, and lower bounds are described in this table. Machine-learning hyperparameter tuning was executed using the "rBayesianOptimization" package[4].

[a] For each tuned hyperparameter, 50 rounds of tuning were performed, capped at a maximum of 200 rounds per model.
[b] Machine-learning references in column 1 also include parameter definitions.
[c] As described in the supplementary methods, the number of features was not tuned for some dimensionality reduction methods.
[d] The maximum possible number of nodes in a decision tree is equal to 2 to the power of the depth. With a depth of 15 in this work, the total maximum number of nodes was 32768.

References

1. Haider SP, Mahajan A, Zeevi T, et al. **PET/CT radiomics signature of human papilloma virus association in oropharyngeal squamous cell carcinoma**. *Eur J Nucl Med Mol Imaging* 2020;
2. Haider SP, Zeevi T, Baumeister P, et al. **Potential Added Value of PET/CT Radiomics for Survival Prognostication beyond AJCC 8th Edition Staging in Oropharyngeal Squamous Cell Carcinoma**. *Cancers (Basel)* 2020;12:
3. Haider SP, Sharaf K, Zeevi T, et al. **Prediction of post-radiotherapy locoregional progression in HPV-associated oropharyngeal squamous cell carcinoma using machine-learning analysis of baseline PET/CT radiomics**. *Transl Oncol* 2020;14:100906
4. Yan Y. rBayesianOptimization: Bayesian Optimization of Hyperparameters. Version 1.1.0 ed; 2016
5. van Griethuysen JJM, Fedorov A, Parmar C, et al. **Computational Radiomics System to Decode the Radiographic Phenotype**. *Cancer Res* 2017;77:e104-e107
6. Pyradiomics-community. Pyradiomics Documentation Release 2.1.2. 2018
7. Friedman JH, Hastie T, Tibshirani R. **Regularization Paths for Generalized Linear Models via Coordinate Descent**. *2010* 2010;33:22
8. Majka M. naivebayes: High Performance Implementation of the Naive Bayes Algorithm in R. Version 0.9.6 ed; 2019
9. Liaw A, Wiener M. **Classification and Regression by randomForest**. *R News* 2002;2:18-22
10. Meyer D, Dimitriadou E, Hornik K, et al. e1071: Misc Functions of the Department of Statistics, Probability Theory Group (Formerly: E1071), TU Wien. Version 1.7-2 ed; 2019
11. Chen T, He T, Benesty M, et al. xgboost: Extreme Gradient Boosting. Version 0.90.0.2 ed; 2019
12. Chen T, Guestrin C. XGBoost: A Scalable Tree Boosting System. *arXiv e-prints*; 2016
